# Potential Action Mechanism and Inhibition Efficacy of *Morinda citrifolia* Essential Oil and Octanoic Acid against *Stagonosporopsis cucurbitacearum* Infestations

**DOI:** 10.3390/molecules27165173

**Published:** 2022-08-13

**Authors:** Mateus S. Dalcin, Bruna L. Dias, Luis O. Viteri Jumbo, Ana C. S. S. Oliveira, Sabrina H. C. Araújo, Wellington S. Moura, Dalmarcia S. C. Mourão, Talita P. S. Ferreira, Fabricio S. Campos, Alex Sander R. Cangussu, Marcos V. G. Alves, Bruno S. Andrade, Javier G. Mantilla-Afanador, Raimundo W. A. Aguiar, Eugênio E. Oliveira, Gil R. Santos

**Affiliations:** 1 Programa de Pós-Graduação Produção Vegetal, Universidade Federal do Tocantins (UFT), Gurupi 77402-970, TO, Brazil; 2 Programa de Pós-Graduação em Biodiversidade e Biotecnologia—Rede Bionorte, Universidade Federal do Tocantins (UFT), Gurupi 77402-970, TO, Brazil; 3 Carrera de Agronomia, Universidad Nacional de Loja (UNL), Loja 110103, Ecuador; 4 Programa de Pós-Graduação Biotecnologia, Universidade Federal do Tocantins (UFT), Gurupi 77410-530, TO, Brazil; 5 Programa de Pós-Graduação Ciências Florestais e Ambientais, Universidade Federal do Tocantins (UFT), Gurupi 77402-970, TO, Brazil; 6 Departamento de Entomologia, Universidade Federal de Viçosa (UFV), Viçosa 36570-900, MG, Brazil; 7 Departamento de Ciências Biológicas, Universidade Estadual do Sudoeste da Bahia, Jequié 45206-190, BA, Brazil; 8 Research Institute in Microbiology and Agroindustrial Biotechnology, Universidad Católica de Manizales, Carrera 23 No. 60-63, Manizales 170002, Colombia

**Keywords:** plant-based biorational fungicides, gummy stem blight, molecular docking, tyrosine–tRNA ligase

## Abstract

The use of plant-based products has been shown to efficiently inhibit fungi-mediated diseases in agricultural crops. Here, we extracted and evaluated the composition of noni, *Morinda citrifolia* L., essential oil and assessed its activities against *Stagonosporopsis cucurbitacearum* in *Cucumis melo* L. Using in silico molecular approaches, potential interactions between the essential oil major components and *S. cucurbitacearum* tyrosine–tRNA ligase were predicted. Finally, we also measured the potential interference of plant physiology (the stomatal conductance and net photosynthesis) mediated by the application of the *M. citrifolia* essential oil. Chromatographic analysis revealed that octanoic acid (75.8%), hexanoic acid (12.8%), and isobutyl pent-4-enyl carbonate (3.1%) were the major essential oil compounds. Octanoic acid and noni essential oil, when used as preventive measures, reduce fungal mycelial growth at a concentration of 5 mg/mL without causing significant damage to the treated leaves, which reinforces their efficacies as preventive tools against *S. cucurbitacearum*. Molecular docking analyses predicted very stable interactions between the major essential oil constituents and *S. cucurbitacearum* tyrosine–tRNA ligase, suggesting the interference of these plant-based molecules upon enzyme activation. Octanoic acid and *M. citrifolia* essential oil at concentrations of 20 mg/mL decreased the stomatal conductance and net photosynthesis rate of melon plants, resulting in robust phytotoxicity. Collectively, our findings indicated that despite the phytotoxicity risks at higher concentrations, *M. citrifolia* essential oil and octanoic acid, have potential as alternative tools for the integrative management of *S. cucurbitacearum*.

## 1. Introduction

The use of plant-based extracts and essential oils (EOs) has shown to be an effective alternative to the use of synthetic compounds for controlling phytopathogens [1,2]. Noni, *Morinda citrifolia* L., a member of the *Rubiaceae* family, is among the plants that produce EOs that can act as potential fungicides without detrimentally affecting beneficial fungi [3,4]. Although previous investigations suggested that the fungicidal activities of *M. citrifolia* essential oil may rely on the actions of its major components—i.e., the octanoic and hexanoic acids [5,6], the confirmation of such statements still requires investigations using other fungal species, and with the emergence of new in silico analysis tools, such as molecular docking, it is possible to define potential targets for the major and minor compounds of EOs [7,8], including those of *M. citrifolia*.

Among the pathogens that attack agricultural crops, fungi have been recognized as the main disease-causing agents, infecting plant tissues at different growth stages, which generally leads to losses in the quality and quantity of agricultural products [9,10]. One of the main pathogens of the plants of the *Cucurbitaceae* family is the fungus *Stagonosporopsis cucurbitacearum* (Fr.) (syn: *Didymella bryoniae*) [11]. This pathogen can infect at least 12 genera and 23 species of *Cucurbitaceae*, including watermelon (*Citrullus lanatus*), cucumber (*Cucumis sativus*), cantaloupe and muskmelon (*Cucumis melo*), squashes (*Cucurbita pepo*), and gourds (*Cucurbita* spp.) [12,13]. Moreover, this fungus is the causal agent of gummy stem blight (GSB) disease, whose main symptoms can be characterized as the falling over of seedlings, circular lesions on the leaves, and canker formation on the stem and tendrils [14,15].

Currently, the application of chemical agents, including fungicides with both specific (difenoconazole, tebuconazole, and thiophanate-methyl) and broad (chlorothalonil and mancozeb) [16,17] modes of action, remains the most commonly used method for GSB control [18]. One of the disadvantages of such a strategy is the fact that these pesticides lose their ability to control the pathogen owing to its increased resistance to these compounds [19,20,21]. In addition, the high frequency of pesticide use has detrimental effects on human health due to its residual effect on food and the environment after reaching the target organism [22,23].

Although alternative options based on natural fungicidal compounds are continuously explored to establish control practices against plant pathogens [24,25], the potential of these natural products for control GSB still is an unresolved task. Thus, the present investigation was conducted aiming to evaluate the efficiency of noni EO and its major compound, octanoic acid, for controlling *S. cucurbitacearum*. By applying in silico approaches, we analyzed the potential molecular interactions of these alternative fungicide products with potential targets of the phytopathogen. Lastly, considering the fact that the misuse of EOs or of the compounds isolated from them can also cause detrimental effects such as phytotoxicity [22,23] and in order to establish acceptable physiological standards of phytotoxicity of noni EO in melon plants, different concentrations were applied, and potential physiological changes in the melon plants were evaluated.

## 2. Results

### 2.1. Chromatographic Analysis of M. citrifolia EO

The ripened fruits yielded an average of 3% of essential oil when extracted by the hydrodistillation method. Chromatographic analyses indicated that *M. citrifolia* EO is mainly composed of carboxylic acids, and octanoic acid (75.8%) and hexanoic acid (12.8%) were the main EO compounds (Table 1). Additionally, isobutyl pent-4-enyl carbonate (3.1%) and 15 other components were identified in the EO sample. The chromatographic profile can be seen in Figure S1.

### 2.2. Interactions of Noni EO Components and Fungal Tyrosine–tRNA Ligases

The identities and validation results (including those for the corresponding Ramachandran-favored values and QMEAN) highlighted the quality of the templates selected for homology modeling (Table 2 and Figure S2).

The major compounds present in noni EO complexed with the fungal tRNA ligase and formed various types of interactions with varying affinity energies (Table 3). The octanoic acid and isobutyl pent-4-enyl carbonate showed better affinity energies (−5.1 kcal/mol) with the target of *S. cucurbitacearum* than other components.

**Figure 1:**
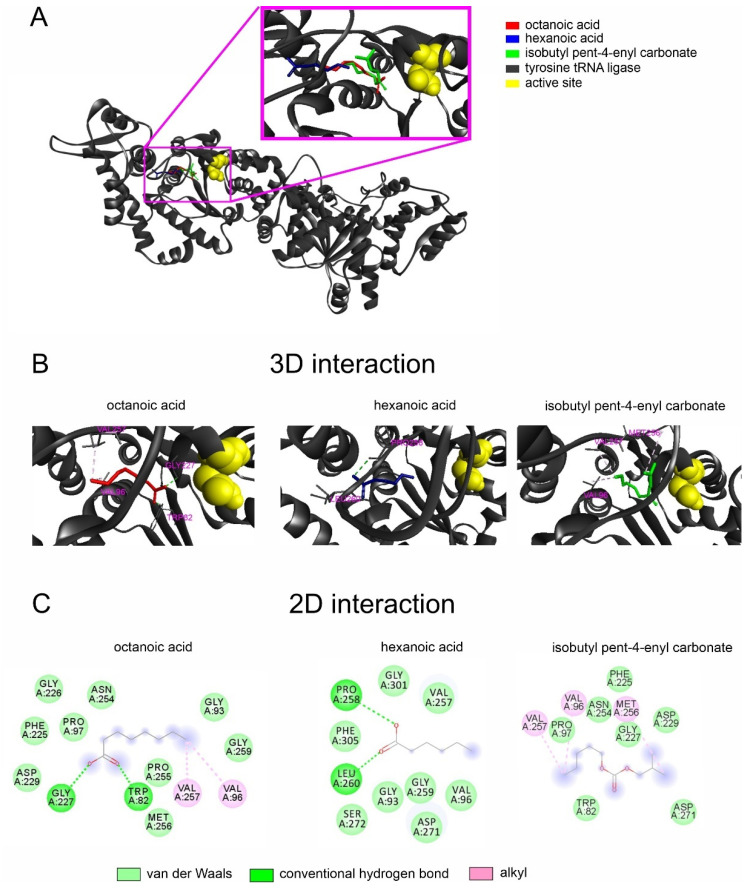
(**A**) Protein structure of *Stagonosporopsis cucurbitacearum* tyrosine-tRNA (gray) and the active site (yellow) interacting with octanoic acid (red), hexanoic acid (blue), and isobutyl pent-4-enyl carbonate (green). (**B**) 3D interaction and (**C**) 2D interaction map representation of *Stagonosporopsis cucurbitacearum* tyrosine-tRNA with octanoic and hexanoic acids and isobutyl pent-4-enyl carbonate. All amino acids belonging to the lipid environment binding site are represented.

The interactions between octanoic acid and the *S. cucurbitacearum* tRNA ligase active site included van der Waals (GLY93, PRO97, PHE225, GLY226, ASP229, ASN254, PRO255, MET256, GLY259), conventional hydrogen bonds (TRP82, GLY227), and alkyl bonds (VAL96, VAL257) (Figure 1). Similar binding patterns were recorded for the complexes formed between butanoic acid, where van der Waals (GLY93, VAL96, VAL257, GLY259, ASP271, SER272, GLY301, PHE305) and conventional hydrogen bonds (PRO258, LEU260) were recorded (Figure 1). Regarding the interactions with isobutyl pent-4-enyl carbonate, our molecular docking revealed van der Waals (TRP82, PRO97, PHE225, GLY227, ASP229, ASN257, ASP271) and alkyl (VAL96, MET256, VAL257) interactions (Figure 1)

In a dynamic molecular simulation environment, the spatial RMSD was calculated from the average position of each amino acid residue of the complexes formed by the ligand octanoic acid, hexanoic acid, and isobutyl pent-4-enyl carbonate with the protein tyrosine-tRNA ligand from *Stagonosporopsis cucurbitacearum* to confirm structural stabilization (Figure S3). The highest values of RMSD were less than 1.0 Å and indicated that the residues that underwent the greatest changes are in the regions corresponding to the loops, not having major conformational changes in the residues of the region of the active site where there is a lower value of RMSD, thus revealing the stability of these areas.

### 2.3. Noni EO and Octanoic Acid Toxicity to S. cucurbitacearum Mycelial Growth and Melon Leaves

**Figure 2:**
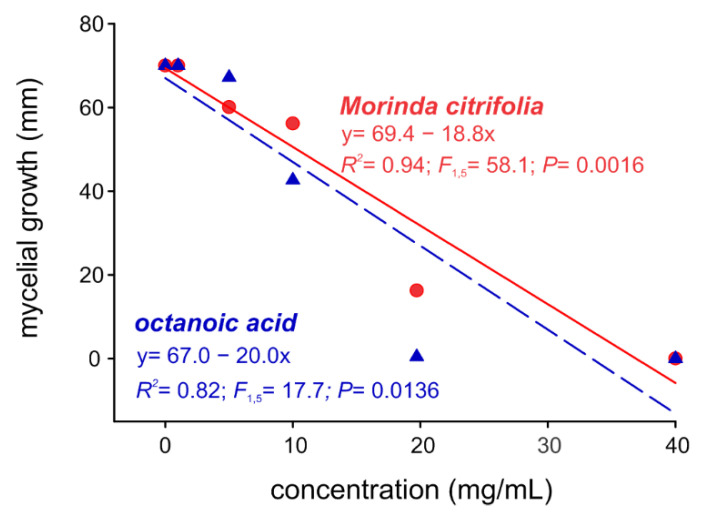
In vitro mycelial growth of the fungus *Stagonosporopsis cucurbitacearum* under the effect of different concentrations of octanoic acid and *Morinda citrifolia* essential oil.

The noni essential oil and octanoic acid similarly inhibited *S. cucurbitacearum* in a concentration-dependent manner (Figure 2). When applied at a concentration of 20 mg/mL, both noni essential oil and octanoic acid almost completely eliminated mycelial growth.

**Figure 3:**
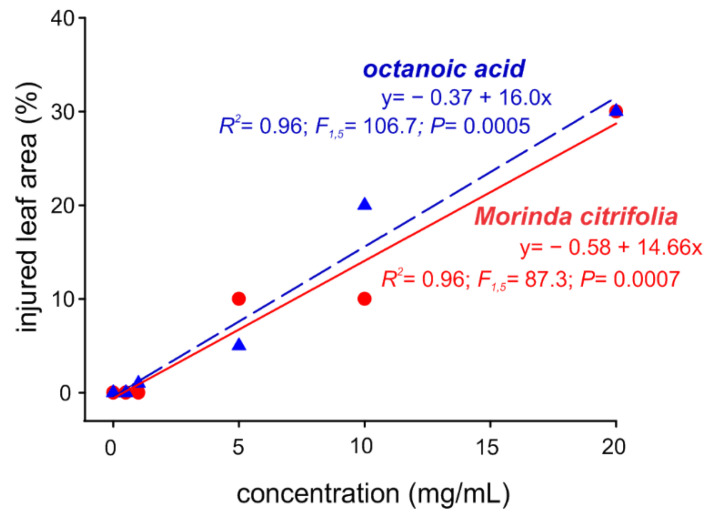
Leaf area damage (phytotoxicity) in *Cucumis melo* plants as a function of different concentrations of the octanoic acid and *Morinda citrifolia* essential oil.

However, at such concentrations, both alternative compounds were highly phytotoxic to melon leaves, causing an injured leaf area of approximately 30% (Figure 3), which led to chlorosis and necrosis. The injured leaf area decreased with the reduction of EO and octanoic acid concentration, where no phytotoxic actions were recorded for concentrations as low as 1 mg/mL (Figure 3).

**Figure 4:**
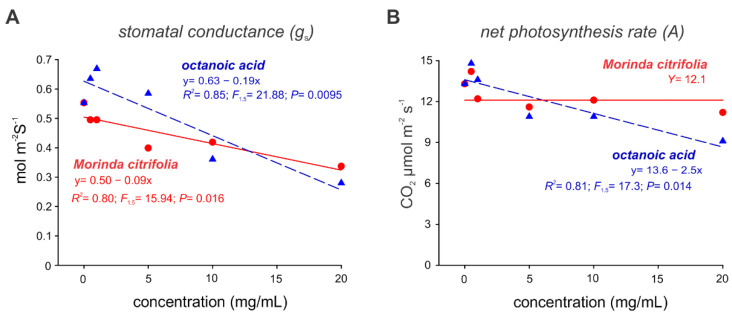
Stomatal conductance (**A**) and net photosynthesis rate (**B**) in *Cucumis melo* plants submitted to the application of octanoic acid and *Morinda citrifolia* essential oil.

Noni EO and octanoic acids seemed to differentially affect the stomatal conductance and net photosynthesis rate of the melon leaves (Figure 4). While increasing concentrations of both of these alternative products reduced stomatal conductance (Figure 4A), only octanoic acid was able to detrimentally affect the photosynthesis rate (Figure 4B).

### 2.4. Evaluation of Preventive Controls of Noni EO in Melon Plants

**Figure 5:**
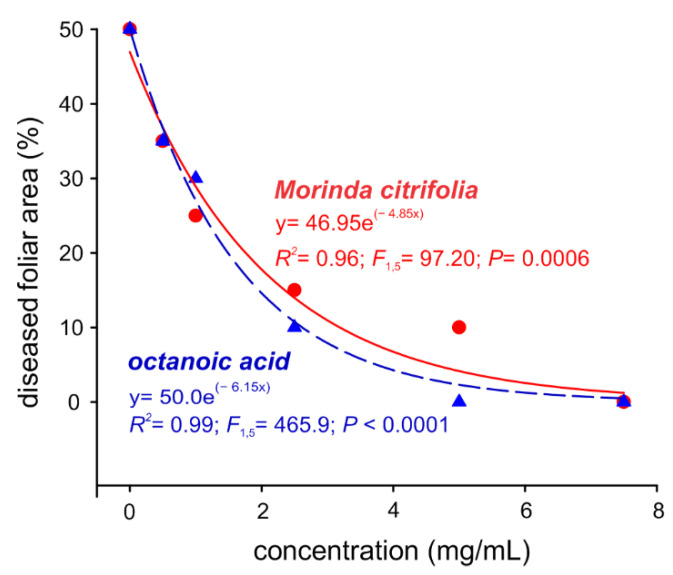
Severity of gummy stem blight in *Cucumis melo* leaves treated with octanoic acid and *Morinda citrifolia* essential oil.

Noni essential oil and octanoic acid exhibited preventive control actions against *S. cucurbitacearum* in melon plants, where DFAs were reduced in a concentration-dependent manner (Figure 5). Leaves treated with concentrations as low as 1 mg/mL, which is the maximum concentration without any phytotoxic effect recorded, showed a diseased foliar area that was 30% lower for both noni essential oil and octanoic acid (Figure 5). Similarly, for leaves treated with a concentration of 5 mg/mL of noni essential oil, which caused phytotoxic damage of less than 10%, GSB development occurred in an area of 10% approximately (Figure 5).

## 3. Discussion

Under in vitro conditions, the essential oil of *M. citrifolia* and its main compound octanoic acid inhibited the mycelial growth of the fungus *S. cucurbitacearum*. This inhibition is due to the three major compounds that show affinities for the enzyme tyrosine–tRNA ligase. Although high concentrations of these products cause physiological changes in treated plants, lower concentrations also prevent gummy blast melon, completely inhibiting disease development without causing phytotoxicity. Therefore, our study reinforces the potential of noni EO and its compounds to inhibit and prevent phytopathogenic diseases in *C. melo*.

Based on the results obtained from phytochemical analysis, the octanoic acids, hexanoic acids, and isobutyl pent-4-enyl carbonate are the main compounds (>91%) in *M. citrifolia* EO, as reported in previous studies [5,6]. However, it was also demonstrated in other investigations [27] that these same compounds represent less than 13% of the EO composition, which shows a strong variation in their chemical composition and consequently in their biological activity. Essential oil bioactivities are generally attributed to their main compounds without neglecting the possible effects of synergism/antagonism with minor compounds [28,29]. Here, we found that *M. citrifolia* EOs completely inhibited the mycelial growth of *S. cucurbitacearum*. Octanoic acid was the most efficient, as it maintained the same effect at half of the concentration. Thus, the fungicide activity can be attributed to this compound. Although there are no reports of the antagonistic effects of octanoic acid and the other compounds identified in this study, negative interactions have been reported between the compounds of EOs when tested with microorganisms, such as fungi [30,31] and bacteria [32].

On the one hand, previous studies have reported bactericidal, fungicidal, and insecticidal effects [4,33,34] of *M. citrifolia* EO, as well as their main compound octanoic acid, [35,36] and fungicidal activities, slowing or inhibiting mycelial growth and conidial germination of the fungi *Penicillium roqueforti*, *Aspergillus niger*, and *Zygosaccharomyces bailii* [37,38,39]. On the other hand, the susceptibility of *S. cucurbitacearum* to EOs has also been reported [40,41].

Although the mechanism of action of EOs in fungi has not been completely elucidated, our molecular docking predicted stable interactions between octanoic acid and the *S. cucurbitacearum* tyrosine–tRNA ligase, indicating the interference of this compound in the enzyme. Similarly, in silico analyses demonstrated the affinity of the main components of noni EO, including octanoic and hexanoic acids, with the tyrosine–tRNA ligase of *Sclerotium rolfsii* [4], suggesting a possible impairment of fungal protein synthesis as a mechanism of fungitoxicity. The authors [42,43] suggested that the damage occurs in the fungal cell envelope, with evident changes in the endomembrane system, particularly in the mitochondria. Likewise, studies by [39] showed that octanoic acid increases the plasma membrane fluidity by physical disruption; when applied to *Saccharomyces* *cerevisiae*, a significant leakage of intracellular material from the cells was observed.

How proteins are simulated that can occur within a simulated time, as molecular results, has been used to study how simulation models occur in a simulated way as simulated results [44]. The complexes formed by the ligands octanoic acid, hexanoic acid, and isobutyl pent-4-enyl carbonate with the protein tyrosine-tRNA ligand from *Stagonosporopsis cucurbitacearum* de showed low values of RMSD in the region of the active site, indicating that the proposed models present good stability in this region, not showing major deviations as proposed by [45].

Here, phytotoxic responses (e.g., the presence of chlorosis followed by necrosis) in melon plant leaves when the essential oil was applied at high concentrations were demonstrated, which can lead to alterations in plant physiological activity. Indeed, despite their efficacy to inhibit fungal infestations, some essential oils and their major constituents have been shown to cause phytotoxic effects when applied at higher concentrations [46,47,48]. These interactions with the physiology of plants have been attributed to their interference with some processes in the plant cells, such as the inhibition of mitosis, decrease in cellular respiration and chlorophyll content, membrane depolarization and ion leakage, removal of cuticular waxes, oxidative damage, and microtubule polymerization [49,50,51]. Although this phytotoxicity is also dependent on the compound [52] and the receptor plants, dicotyledonous species are more susceptible than monocotyledonous plants [53,54].

Stomata play a key role in plants as they control both water loss and CO_2_ uptake, and their function can be altered by EO compounds to increase the stomatal aperture [55,56] or induce a total closure [51] with lethal effects on the plants. This relationship is measured by stomatal conductance (mmol m^−2^ s^−1^), which in turn is correlated with the rate of photosynthesis [57,58]. Here, we observed a decrease in the stomatal conductance and photosynthesis rates depending on the applied concentrations, which was most evident in octanoic acid. The decrease in stomatal conductance affects the ability of a plant to perform photosynthesis by decreasing the gas passage through the stomata, and the amount of carbon available for fixation is reduced, thus decreasing the plant photosynthetic rate [59,60]. The evaluation of the relationship between photosynthetic rates, leaf conductivity of CO_2_, and yield in different melon genotypes showed that in fact those genotypes that presented higher photosynthetic rates had higher yields [61].Therefore, adjustment of phytotoxicity-acceptable changes to parameters caused by alternative treatments should be considered so that the productivity of the crop is not compromised.

Treatment strategies are expected to inhibit or control the growth of pathogens, without harming the host plants. Here, phytotoxicity was observed at both EO and various octanoic acid concentrations, thereby limiting their application in the field. However, when applied at lower concentrations and as a preventive measure for GSB control in young (15 days after emergence) melon plants, these products prevented the colonization of *S.* *cucurbitacearum*. Such statements need to be taken carefully into consideration, as we only used young melon plants and did not apply the essential oil during other plant phases (e.g., blooming, fruitification, fruit ripening). However, considering the fact that it is not common, under Brazilian Cerrado biome conditions, the occurrence of GSB symptoms in melon fruits, because it is a disease of leaves and stems, the use of noni EO to control such a phytopathogen in other phases would address objectives other than those aimed here. Collectively, the findings described here demonstrate that noni EO and octanoic acid can be used as alternative tools to prevent the growth of *S. cucurbitacearum* under field conditions.

## 4. Materials and Methods

### 4.1. EO Extraction and Chemical Analysis

We collected ripe *M. citrifolia* plants cultivated in a pesticide-free environment in Gurupi County (11°43′4′′ S, 49°04′07′′ W, Gurupi, TO, Brazil). The samples were deposited in the herbarium located in Universidade Estadual do Tocantins-UNITINS, Brazil, under the identification number 8035. The EO was obtained by the hydrodistillation method, following previously described procedures [4]. Briefly, 500 g of ripe fruit and 500 mL of distilled water were placed in a 1000 mL round-bottom flask and then coupled to a Clevenger apparatus. After a 2-h extraction period, EO was collected in an amber bottle and stored at 4 °C. Octanoic acid was purchased from a commercial chemical company (Sigma–Aldrich^®^, São Paulo, SP, Brazil). We identified and quantified the EO constituents by conducting gas chromatography coupled to mass spectrometry (GC–MS), following previously described procedures [3].

The chromatograph used was the Shimadzu model GC-210 equipped with a selective mass detector model QP2010 Plus operated under the following conditions: RTX-5MS fused silica capillary column (30 m × 0.25 mm × 0.25-μm-thick film); temperature programming 60–240 °C (3 °C/min); injector temperature 220 °C; helium carrier gas; splitless injection with an injected volume of 1 μL of a 1:1000 solution in hexane. For the mass spectrometer, we used an impact energy of 70 eV and an ion source and interface temperature of 200 °C. The spectra were compared with the Nist and Wiley 229 library database and the retention index, calculated for each constituent, and compared with tabulated values, according to [26], and the quantification of the contents of the compounds was expressed as a percentage.

### 4.2. In Silico Studies of the Interactions among Noni EO Major Compounds and the Fungal Tyrosine–tRNA Ligase

#### 4.2.1. Ligands and Modeling Targets

We constructed 3D structures of EO major compounds (i.e., octanoic acid, hexanoic acid, and isobutyl pent-4-enyl carbonate) in their neutral forms, using Marvin Sketch 18.10, ChemAxon software (accessed on 4 March 2022 at http://www.chemaxon.com).

Amino acid sequences of tyrosine–tRNA ligase from *S. cucurbitacearum* were obtained from previous investigations [62]. The protein 3D structure was constructed using a homology modeling approach with the Swiss Model Workspace (accessed on 13 April 2022 at https://swissmodel.expasy.org/) after the selection of its respective templates using the Protein Basic Local Align Search Tool. Templates were downloaded from the Protein Databank (accessed on 13 April 2022 at https://www.rcsb.org/), and the experimental method, resolution, R-value, and its complexing with a ligand were taken as the quality parameters. We used the Swiss model to check the protein structure crashes and amino acid positioning in the active sites, as previously suggested by [63]. By inspecting the Ramachandran plots [64,65], we validated the generated models and analyzed the distribution of the torsion angles of the backbone Φ and ψ, which are responsible for the stereochemical quality of the proteins studied as well as the QMEAN factor [66].

#### 4.2.2. Molecular Docking Calculations

We prepared the targets and ligands using molecular docking processes available at Autodock Tools 1.5.7 [67], according to the methodology proposed elsewhere [68]. We used AutoDock Vina [69] in the docking calculations, which resulted in nine docking positions for ligand–target interactions, which provided the affinity energy values (kcal/mol). To select the best position for each ligand inside the protein target, we analyzed the docking position results using PyMOL 2.0 Schrödinger, LLC (San Carlos, USA) [70] and Discovery Studio 4.5 Dassault Systemes BIOVIA (Vélizy-Villacoublay, France) [71], as previously proposed by [68].

#### 4.2.3. Molecular Dynamics Simulation

Molecular dynamics simulations were performed using the MDWeb server [72], and the molecular docking PDB files were used to prepare the simulation base. The structure was prepared using the Gromacs full MD Setup with the AMBER-99SB * force field, as it satisfactorily describes the molecular behavior of proteins. The molecular dynamics simulation process was carried out according to the set of steps that include cleaning the structure, fixing the side chains of the complex, adding the solvent box, minimizing energy, and balancing the system to receive the minimized structure as an outlet [45]. These simulations were performed in constant volume (NVT) [73,74]. In the equilibrium stage, the systems were subjected to a simulation of 2.5 ps with a temperature of 300 K and constant pressure. After the generation of the protein–ligand complex, the water molecules and ions were removed to reduce the size of the system, and the dry trajectory was recovered to trace the mean quadratic deviation (RMSD).

### 4.3. In Vitro Activities of Noni EO against S. cucurbitacearum

Bioassays were performed in commercial potato-dextrose-agar culture medium contained in Petri dishes (70 mm in diameter). Five concentrations (1, 5, 10, 20, and 40 mg/mL) of noni EO and octanoic acid were diluted in a mixture of distilled water and 1.0 % (*v*/*v*) Tween 20 (Êxodo Científica, Hortolândia, São Paulo, Brazil). The test solutions were applied (at a volume of 200 µL) to cover the inner surfaces of Petri dishes (9 cm in diameter). Disks (4 mm in diameter) containing fungal mycelium were added to the center of each Petri dish and placed in an incubation chamber at 25 °C for 10 d. Four replicates were used for each compound and concentration combination. As a positive control, we used a commercial formulation containing a mixture of synthetic fungicides, thiophanate-methyl (200 g/kg), and chlorothalonil (500 g/kg) at an application rate of 2 g/L, and the negative control consisted of distilled water–Tween 80 (1.0% *v*/*v*).

### 4.4. Toxicity and Physiological Parameters of the Melon Plants Treated with Noni EO

Melon, *Cucumis melon* L., plants of the cultivar Eldorado 300, were individually cultivated in pots (5 L) filled with a commercial substrate (Germinar XI; Bioflora Ltd.a, Prata, MG, Brazil) and sieved soil in a greenhouse environment. Then, 15 d after emergence, plants were sprayed up to the leaf drip point with EO-containing solutions at concentrations of 0.5, 1, 5, 10, and 20 mg/mL. Plants were kept under controlled conditions (25 °C, 70 ± 5% humidity) for 24 h to evaluate their phytotoxic actions. We used the phytotoxicity scale adapted from [75]: 1–25% = slight leaf or plant chlorosis; 26–50% = moderate leaf or plant chlorosis; 51–75% = high leaf or plant chlorosis; 76–100% = plants wilt and die. We used five plants (replicates) for each fungicide (EO and octanoic acid) and concentration combination. The control treatment consisted of plants sprayed with distilled water and Tween (1% *v*/*v*) solutions.

We further evaluated whether noni EO and octanoic acid would interfere with the melon plant physiology (e.g., stomatal conductance and gas exchange rates). Stomatal conductance (*g*_l_; mol m^−2^ s^−1^) and CO_2_ net assimilation (*A*; μmol m^−2^ s^−1^), which is a photosynthesis indicator, were measured under environmental CO_2_ conditions using a portable infrared gas analyzer (model LI-6400 XT, Li-color, Inc. Lincoln, Dearborn, MI, USA). The leaf chamber was adjusted to work under artificial light saturation of 1200 mmol m^−2^ s^−1^, the internal temperature of the chamber was adjusted to 30 °C, and the CO_2_ concentration was standardized to that in the environment.

### 4.5. Preventive Control of S. cucurbitacearum by Noni EO

The melon plants used and the fungicide (noni EO and octanoic acid) treatments were similar to those described in the phytotoxicity bioassay section. Briefly, 30 days after emergence, plants were sprayed up to the leaf drip point, and after a 2-h period, we inoculated the pathogen into the leaves. We used disks (5 mm in diameter) containing *S. cucurbitacearum* mycelium with the aid of a sterilized pin for fixation. The plants were then kept in a humid and dark chamber for 48 h. Subsequently, the melon plants were placed in a natural environment with a temperature ranging from 24 °C to 34 °C to allow disease development. Seven days after inoculation, the disease severity was evaluated using the grading scale adopted by [76]: 0 = healthy plant; 1 = diseased foliar area (DFA) less than 1%; 3 = 1% to 5% DFA; 5 = 6% to 25% DFA; 7 = 26% to 50% DFA; 9 = DFA greater than 50%.

### 4.6. Statistical Analysis

The results of the mycelial growth inhibition, phytotoxicity, photosynthesis, stomatal conductance, and curative actions of noni EO and octanoic acid were subjected to regression analysis using the curve-fitting procedures available in SigmaPlot 12.5 software (Systat Software Inc., San Jose, CA, USA). The regression model was chosen based on parsimony, and the assumptions of normality and homogeneity of variance were also checked.

## 5. Conclusions

*Morinda citrifolia* essential oil and its major constituent, octanoic acid, were effective in the control of *S. cucurbitacearum* mycelial growth. Although phytotoxic effects in melon plants were recorded when these compounds were applied at 20 mg/mL, lower concentrations of *M. citrifolia* essential oil and octanoic acids were effective in preventing *S. cucurbitacearum* infections without affecting these plants‘ physiology. By using in silico approaches, the involvement of tyrosine–tRNA ligase as a potential target for *M. citrifolia* essential oil constituents, especially octanoic acid was demonstrated. Together, the findings described here highlight the potential of plant-based biorational products to be integrated into the management of *S. cucurbitacearum* in melon fields.

## Figures and Tables

**Table 1:** Chemical composition of *Morinda citrifolia* essential obtained by gas chromatography coupled to mass spectrometry (GC-MS).

Peak	Compounds	RT ^a^ (min)	RI ^b^	RI ^c^	Peak Area (%)	Chemical Class	CAS Number
1	2-hexanone,5-methyl-	3.557	3.667	3.525	0.22	Ketone	110-12-3
2	Hexanoic acid, methyl ester	3.973	4.125	3.933	1.27	Fatty acid	106-70-7
3	Hexanoic acid	5.113	5.550	4.800	12.75	Fatty acid	142-62-1
4	Benzene, tert-butyl-	5.454	5.542	5.383	0.08	Aromatic hydrocarbon	98-06-6
5	Butanoic acid,4-pentenyl ester	6.012	6.125	5.983	0.19	Fatty acid	30563-31-6
6	2-Hexanone, 5-methyl-	6.417	6.500	6.375	0.03	Ketone	110-12-3
7	(E)-2-Methylbut-2-en-1-yl isobutyrate	6.537	6.633	6.500	0.05	Ester	95654-17-4
8	Octanoic acid, methyl ester	6.363	7.058	6.825	2.91	Fatty acid	111-11-5
9	Cyclopropane,1,2,3-trimethyl-	7.333	7.408	7.242	0.07	Hydrocarbon	42984-19-0
10	Octanoic acid	8.130	8.642	7.625	75.77	Fatty acid	124-07-2
11	Citronellol	8.442	8.508	8.417	0.03	Monoterpenoid	106-22-9
12	Hexanoic acid, 4-pentenyl ester	8.861	9.008	8.767	2.57	Fatty acid	30563-33-8
13	1-Pentene, 5-(pentyloxy)-	9.319	9.392	9.250	0.37	Ether	56052-88-1
14	Pentane, 2,2′-oxybis-	9.425	9.467	9.392	0.05	Ether	56762-00-6
15	Decanoic acid, methyl ester	9.719	9.783	9.683	0.15	Fatty acid	110-42-9
16	Hexanoic acid, hexyl ester	10.559	10.617	10.508	0.07	Fatty acid	6378-65-0
17	Isobutyl pent-4-enyl carbonate	11.490	11.825	11.433	3.12	Carbonate	0-00-0
18	Dodecanoic acid, 2-penten-1-yl ester	11.977	12.208	11.825	0.31	Fatty acid	0-00-0
Total					100		

^a^ Retention time, ^b^ Retention indices calculated on an RTX-5MS column, ^c^ Retention indices according to the literature [26].

**Table 2:** Model of *Stagonosporopsis cucurbitacearum* t-RNA ligase used to analyze the molecular docking with the noni essential oil major compounds.

Organism	Target	PDB Template	Identity (%)	Ramachandran Favored (%)	QMEAN
*Stagonosporopsis cucurbitacearum*	Tyrosine—tRNA ligase(EVM0001193.1) *	5THH	52.7%	93.97%	0.74

* BioProject (PRJNA694739) in the National Center for Biotechnology Information (NCBI).

**Table 3:** Molecular docking results for complexes between noni essential oil major compounds and *Stagonosporopsis cucurbitacearum* t-RNA ligase.

Organism	Ligand	Affinity Energy (kcal/mol)
*Stagonosporopsis cucurbitacearum*	Octanoic acid	−5.1
	Isobutyl pent-4-enyl carbonate	−5.1
	Hexanoic acid	−4.7

## Data Availability

Not available.

## Supplementary Materials

The following are available online at https://www.mdpi.com/article/10.3390/molecules27165173/s1, Figure S1: Chromatographic profile from *Morinda citrifolia* essential oil. Figure S2: Homology data from (A) Ramachandran plots, (B) QMEAN plots, and (C) sequence alignment. Figure S3. Dynamic simulation graph of the molecular representation of the structural deviation of RMSD by treatment of the complex formed by the ligand (A) octanoic acid, (B) hexanoic acid, and (C) isobutyl pent-4-enyl carbonate and without ligand (D) with the protein tyrosine-tRNA ligand from *Stagonosporopsis cucurbitacearum*.
