# Supplementary material for: Potential Action Mechanism and Inhibition Efficacy of Morinda citrifolia Essential Oil and Octanoic Acid against Stagonosporopsis cucurbitacearum Infestations
Source: Molecules. 2022 Aug 13;27(16):5173. doi: 10.3390/molecules27165173 (PMC9414982; doi:10.3390/molecules27165173)
Supplement: Supplementary file 1 [file molecules-27-05173-s001.zip › molecules-1809402-supplementary.pdf]

molecules

MDPI

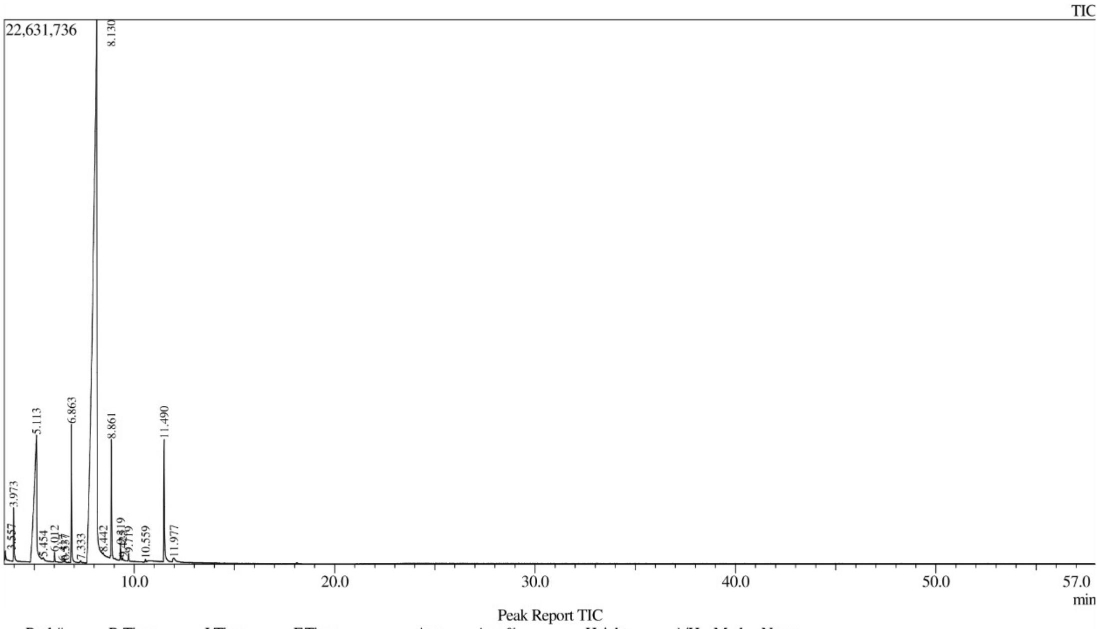

Peak Report TIC

Peak#	R.Time	I.Time	F.Time	Area	Area%	Height	A/H	Mark	Name
1	3.557	3.525	3.667	920375	0.22	390398	2.36		2-Hexanone, 5-methyl-
2	3.973	3.933	4.125	5242636	1.27	2204451	2.38		Hexanoic acid, methyl ester
3	5.113	4.800	5.550	52705876	12.75	5266099	10.01	S	Hexanoic acid
4	5.454	5.383	5.542	342625	0.08	81995	4.18	T	Benzene, tert-butyl-
5	6.012	5.983	6.125	782346	0.19	393041	1.99		Butanoic acid, 4-pentenyl ester
6	6.417	6.375	6.500	128654	0.03	30508	4.22		2-Hexanone, 5-methyl-
7	6.537	6.500	6.633	205154	0.05	62482	3.28	V	(E)-2-Methylbut-2-en-1-yl isobutyrate
8	6.863	6.825	7.058	12028561	2.91	5765950	2.09		Octanoic acid, methyl ester
9	7.333	7.242	7.408	280034	0.07	83369	3.36		Cyclopropane, 1,2,3-trimethyl-
10	8.130	7.625	8.642	313235480	75.77	22447141	13.95	S	Octanoic acid
11	8.442	8.417	8.508	106704	0.03	60214	1.77	T	Citronellol
12	8.861	8.767	9.008	10641349	2.57	4947097	2.15		Hexanoic acid, 4-pentenyl ester
13	9.319	9.250	9.392	1524377	0.37	633044	2.41		1-Pentene, 5-(pentyloxy)-
14	9.425	9.392	9.467	194217	0.05	96594	2.01	V	Pentane, 2,2'-oxybis-
15	9.719	9.683	9.783	608655	0.15	314920	1.93		Decanoic acid, methyl ester
16	10.559	10.508	10.617	293671	0.07	110365	2.66		Hexanoic acid, hexyl ester
17	11.490	11.433	11.825	12902467	3.12	5105572	2.53		Isobutyl pent-4-enyl carbonate
18	11.977	11.825	12.208	1271658	0.31	156361	8.13	V	Dodecanoic acid, 2-penten-1-yl ester
				413414839	100.00	48149601			

Figure S1. Chromatographic profile from *Morinda citSifolia* essential oil.

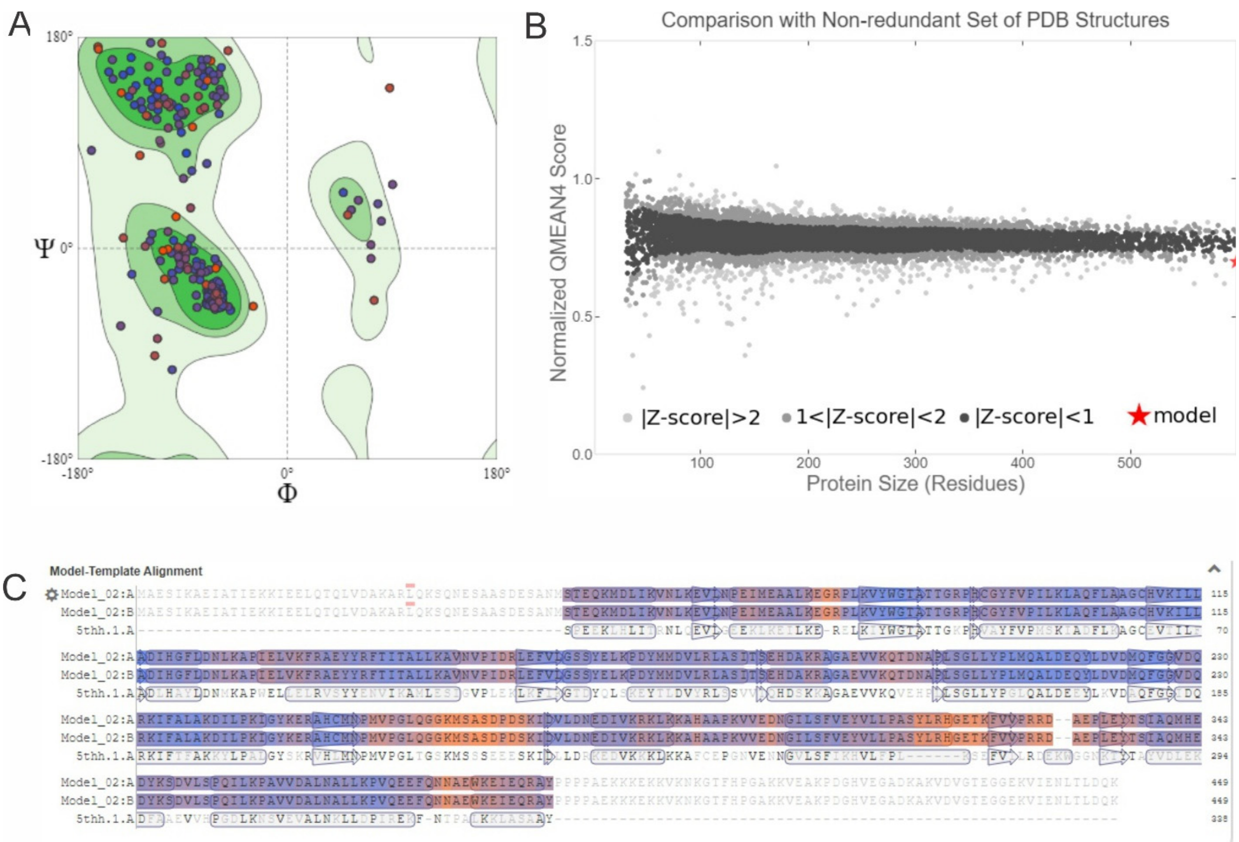

Figure S2. Homology data from (A) Ramachandran plots, (B) QMEAN plots, and (C) sequence alignment.

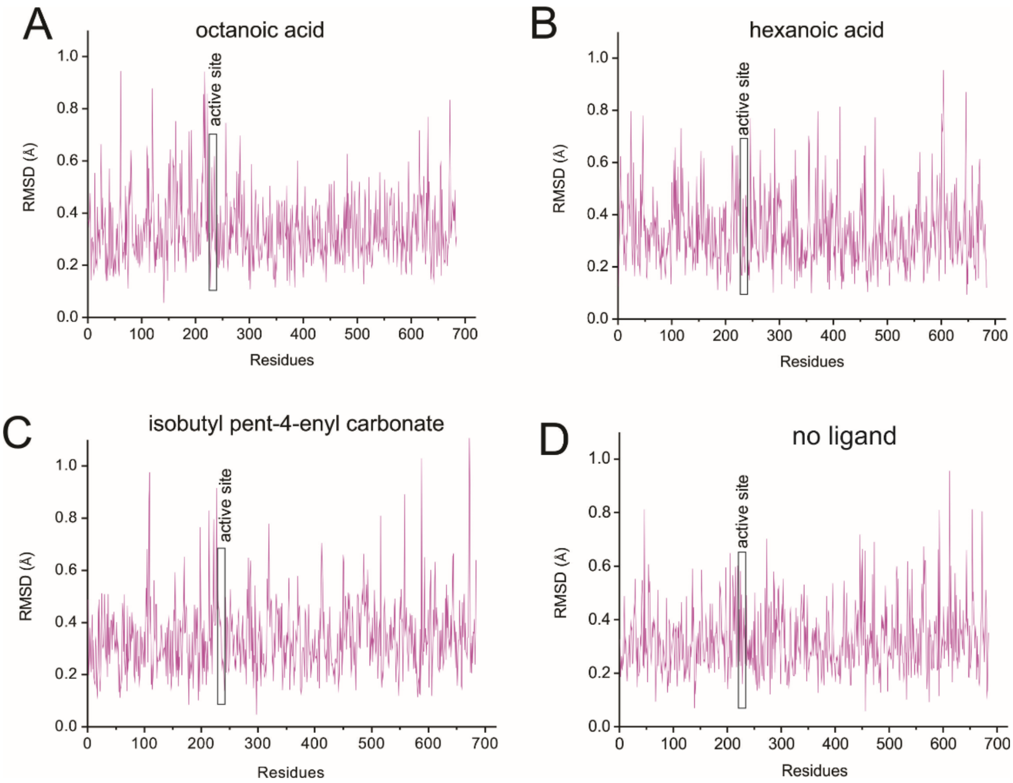

Figure S3. Dynamic simulation graph of the molecular representation of the structural deviation of RMSD by treatment of the complex formed by the ligand (***A***) octanoic acid, (***B***) hexanoic acid, and (***C***) isobutyl pent-4-enyl carbonate and without ligand (***D***) with the protein tyrosine-tRNA ligand from *Stagonosporopsis cucurbitacearum*.
